# Incidence, Risk Factors, and Clinico-Radiological Correlations of Early Post-traumatic Seizures: A Prospective Analysis at a Tertiary Care Center in India

**DOI:** 10.7759/cureus.108519

**Published:** 2026-05-08

**Authors:** Rohini Chaudhari, Sourabh Zambre, Nitish Kumar, Mayur Ingle, Dhiraj Asole

**Affiliations:** 1 Radiology, Indira Gandhi Government Medical College, Nagpur, Nagpur, IND; 2 Neurosurgery, All India Institute of Medical Sciences, Nagpur, Nagpur, IND; 3 Neurological Surgery, Dr. Ram Manohar Lohia Hospital, New Delhi, IND; 4 Neurological Surgery, All India Institute of Medical Sciences, Nagpur, Nagpur, IND

**Keywords:** ct scan head, early post-traumatic epilepsy, head injury, post-traumatic seizures, traumatic brain injury

## Abstract

Background

Post-traumatic epilepsy (PTE) is a major cause of acquired epilepsy. Early post-traumatic seizures (EPTS), occurring within the first seven days following traumatic brain injury (TBI), are known to worsen secondary brain damage and adversely affect patient outcomes. Despite the recognized burden, the incidence and risk factors of EPTS in the Indian population remain poorly characterized.

Objective

To determine the incidence of EPTS and identify the associated demographic, clinical, and radiological risk factors in patients presenting to a tertiary care neurosurgical institute.

Methods

This prospective observational study was conducted over two years (November 2019-October 2021) at the Department of Neurosurgery of a tertiary care teaching institute in India. A total of 1,035 consecutive patients with head injuries who presented to the emergency room were enrolled. Patients were started on levetiracetam 500 mg 12 hourly in adults and 20mg/kg 12 hourly in pediatric patients, in oral or intravenous (I.V.) route as per clinical assessment. The decision to start antiepileptics was clinical; not all patients received antiepileptics. However, a positive CT finding, prior history of seizures, or a Glasgow Coma Scale (GCS) of less than 13 were started on anti-epileptics as per the institute protocol. Patients were monitored for clinical seizure activity during the first seven days post-injury or until hospital discharge, whichever occurred first. Patients were classified according to the GCS score, mechanism of injury, CT findings, age, and sex.

Results

EPTS occurred in 29 (2.8%) of the 1,035 patients. Seizure incidence was highest in the severe TBI group (GCS 3-6: 5 (35.9%)), followed by moderate TBI (GCS 7-12: 11 (9.0%)), and mild TBI (GCS 13-15: 13 (1.5%)). Falls were the most common mechanism, with the highest seizure rate (15, 4.09%). The CT findings associated with the highest EPTS risk included brainstem contusion (1, 50%), subdural hemorrhage (8, 17.7%), and hemorrhagic contusion (7, 10%). The 0-10 age group had the highest absolute number of seizures (13, 6.2%). Male patients accounted for 76.7% (n=22) of the EPTS cases.

Conclusions

The incidence of EPTS in this study was 2.8%. The severity of TBI (p <0.001), a positive hemorrhagic finding in CT scan (p <0.001), and a younger age group (0-10 years) (p = 0.036) were statistically significantly associated with EPTS. These findings emphasize the need for targeted seizure surveillance and prophylaxis in high-risk TBI subgroups.

## Introduction

Early post-traumatic seizures (EPTS) have been recognized as a serious complication of head injury since antiquity. Hippocrates documented seizures following head injury as markers of poor prognosis, and the relationship between traumatic brain injury (TBI) and delayed epilepsy was formally described in the 14th century. Today, TBI is recognized as a major etiological factor in epilepsy, responsible for approximately 5.5% of all cases [1].

Post-traumatic epilepsy (PTE), defined as recurrent seizures arising as a chronic consequence of TBI, accounts for approximately 20% of symptomatic epilepsy in the general population and 5% of cases referred to specialized epilepsy centers [2,3]. In military cohorts, the incidence of PTE rises to 50% owing to the high prevalence of penetrating head wounds [4]. Among civilian patients with TBI, the incidence of immediate seizures ranges from 1% to 4%, EPTS from 4% to 25%, and late post-traumatic seizures from 9% to 42% [3,4].

EPTS is defined as seizures occurring within the first seven days of injury. They are considered direct reactions to acute brain damage rather than manifestations of chronic epileptogenesis [5,6]. EPTS can worsen secondary brain injury by increasing intracranial pressure, exacerbating cerebral ischemia, and elevating metabolic demands. Approximately 80% of individuals with PTE experience their first seizure within 12 months of injury, and more than 90% experience seizures by the end of the second year [7].

Established risk factors for EPTS include prolonged loss of consciousness, missile and penetrating injuries, intracerebral hemorrhage, diffuse cerebral contusions, post-traumatic amnesia lasting more than three days, acute subdural hematoma requiring surgical evacuation, depressed skull fracture, and early seizure [2,8]. Brain contusions and subdural hematomas confer the highest risk of late seizures, which persists for up to 20 years [3]. A substantial body of literature has examined EPTS incidence and risk factors across diverse populations, with considerable variability across studies in terms of patient selection, injury severity classification, and follow-up duration.

Jennett (1950s, n=1,000, Oxford) reported that 5% of hospitalized head injury patients developed one or more fits within the first week, establishing an early epidemiological baseline [1]. Annegers et al. (1935-1984, n=4,541, Minnesota) identified brain contusion and subdural hematoma as the strongest predictors of late seizures, with skull fractures and prolonged loss of consciousness as weaker but significant risk factors [2].

Englander et al. (n=647, multicenter United States, 1993-1997) found a 3% incidence of EPTS and identified biparietal contusions, dural penetration with bone and metal fragments, multiple intracranial operations, midline shift greater than 5 mm, and bilateral cortical contusions as predictors of the highest cumulative probability for late PTS [9].

Wiedemayer et al. (n=1,868, Essen, Germany, 1988-1998) reported a 5.8% overall EPTS incidence, with chronic alcohol abuse as the single strongest risk factor, followed by subdural hematoma and brain contusion. Notably, the risk of EPTS was higher in moderate than in severe head injury [10].

In pediatric populations, Liesemer et al. (n=275, United States) found a 12% EPTS incidence within the first seven days of injury. Younger age (<2 years), severe TBI (Glasgow Coma Scale (GCS) <8 post-resuscitation), prehospital hypoxia, and impact seizures were independently associated with EPTS [11]. Ong et al. (n=966 children, Malaysia) reported a 5.5% incidence with female sex, age under two years, loss of consciousness exceeding 24 hours, and acute subdural hematoma as significant predictors [12].

Asikainen et al. (n=490, Finland) documented an overall EPTS incidence of 16.9%, with children aged seven years or younger most prone to early seizures (30.8%) [13]. Lee et al. (Taiwan) reported EPTS incidence of 2.36% in mild, 4.1% in moderate, and 3.6% in severe closed head injury cohorts, with the majority of seizures being generalized tonic-clonic type [14,15].

Oluwole (n=266, Nigeria) demonstrated that the severity of TBI, measured by combined neurological and neuroimaging features, is a better predictor of EPTS than GCS alone and found that subjects younger than 12 years were eight times more likely to develop EPTS than those over 50 years [16].

Collectively, these studies demonstrate that EPTS incidence varies significantly by population (1-17%), that younger age and structural brain injury consistently emerge as key risk factors, and that sex (male predominance) and mechanism of injury contribute meaningfully to risk stratification.

Despite this global burden, population-specific data from Indian tertiary care centers are limited. This study was designed to prospectively determine the incidence and risk factors of EPTS among patients with head injury presenting to a tertiary neurosurgical center in India.

Pathophysiology

The mechanisms underlying PTE are multifactorial and differ between immediate/early and late-onset seizures. Immediate and early seizures are believed to represent direct reactions to acute brain damage, whereas late seizures result from chronic epileptogenic remodeling [5,6].

Closed head injuries produce diffuse axonal injury, cerebral edema, and ischemia, leading to the release of excitatory amino acids, cytokines, bioactive lipids, and toxic mediators that cause secondary cellular damage [17]. Penetrating injuries create cortical cicatrix formation, associated with an approximately 50% risk of PTE [4]. Non-penetrating injuries with focal contusions or intracranial hemorrhages carry up to a 30% PTE risk, potentially mediated by the toxic effects of hemoglobin degradation products on neuronal function [18].

Following hemorrhagic cortical injury, ferrous compounds are deposited in the neural tissue, initiating Haber-Weiss iron-catalyzed reactions that generate hydroxyl radicals, triggering peroxidation of phospholipid membranes and cell death [19]. Iron liberated from hemoglobin and hemoglobin itself generate reactive oxygen species (ROS) and reactive nitrogen species (RNS), both of which are implicated in seizure mechanisms [20]. Excessive activation of excitatory neurotransmitter receptors generates nitric oxide and ROS, accelerating the production of neurotoxic guanidino compounds in a self-reinforcing cycle [21]. This imbalance, characterized by increased excitatory amino acid release (e.g., aspartic acid) and decreased inhibitory amino acid activity (e.g., gamma-aminobutyric acid (GABA)), triggers excitotoxicity at N-methyl-D-aspartate (NMDA) receptors and may establish a chronic epileptogenic focus [22].

Regarding prophylactic anticonvulsants, phenytoin and carbamazepine have demonstrated efficacy in reducing early PTE in high-risk patients; however, routine prophylaxis with these agents does not prevent late post-traumatic seizures and is not recommended beyond the first seven days [23]. Current evidence does not support the notion that preventing early seizures reduces mortality, morbidity, or the development of late PTE [24].

## Materials and methods

Study design and setting

This prospective observational study was conducted at the Department of Neurosurgery at a tertiary care teaching institute in India. The study period was from November 2019 to October 2021. Patients with a prior history of seizures, presentation post seven days of injury, previous cranial surgery, death within 24 hours, known electrolyte disturbances, and chronic kidney disease were excluded.

Participants

**Figure 1:**
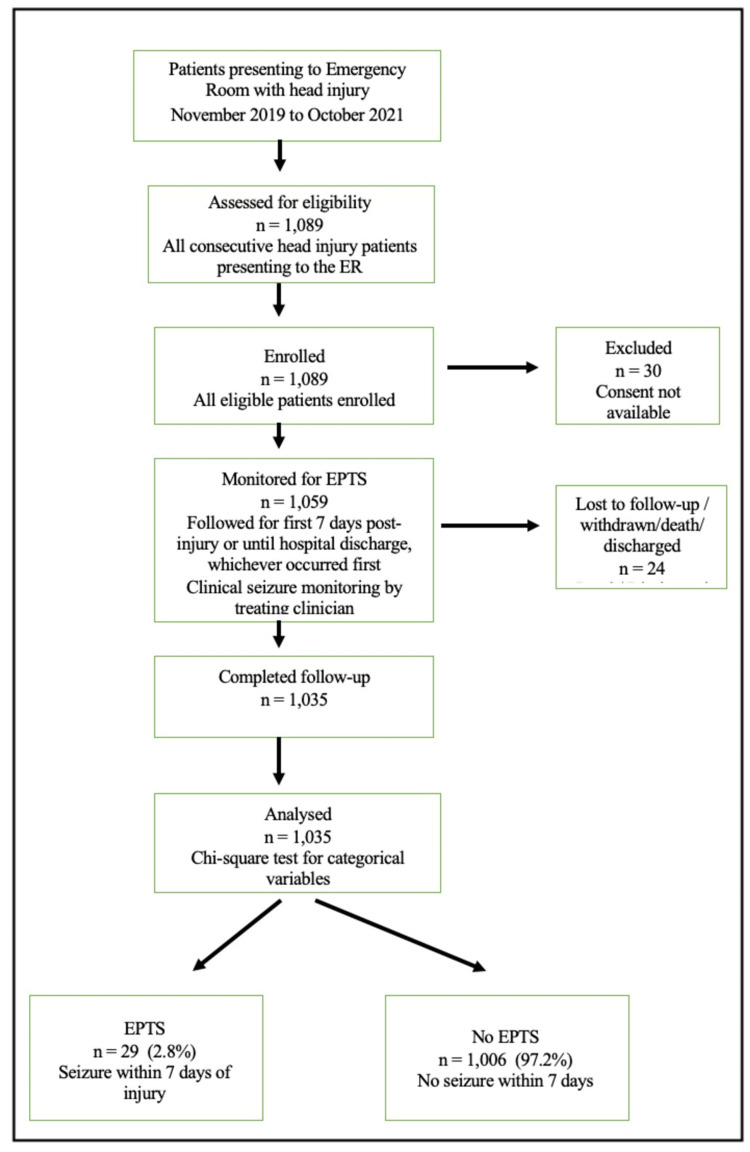
STROBE diagram illustrating the enrollment pattern of the study participants. STROBE: STrengthening the Reporting of OBservational studies in Epidemiology; EPTS: early post-traumatic seizures; ER: emergency room

All patients presenting to the emergency room with a diagnosis of head injury during the study period were screened for eligibility. A total of 1,089 consecutive patients were enrolled in the study. Thirty patients were excluded as per the exclusion criteria. Further, 24 patients could not be analyzed due to various factors like withdrawal from the study, discharge against medical advice, and death. Analysis was done on 1,035 patients, as some were excluded, as shown in Figure 1. Patients were started on levetiracetam 500 mg 12 hourly in adults and 20 mg/kg 12 hourly in pediatric patients, in oral or intravenous (I.V.) route as per clinical assessment. The decision to start antiepileptics was clinical; not all patients received antiepileptics. However, a positive CT finding, prior history of seizures, or a GCS of less than 13 were started on anti-epileptics as per the institute protocol. Patients were monitored from the time of admission until the first seven days post-injury or hospital discharge, whichever came first.

Seizure definition

Post-traumatic seizure was defined as a discrete clinical seizure event following TBI, confirmed by clinician documentation of rhythmic extremity or facial movement, autonomic instability, or other characteristic seizure manifestations not attributable to another obvious cause. EPTS was defined as a seizure occurring within the first seven days of injury.

Classification of TBI severity

TBI severity was classified based on the GCS score as severe (GCS score 3-8), moderate (GCS score 9-12), or mild (GCS score 13-15). For the analysis of seizure incidence, patients were sub-grouped as GCS 3-6, GCS 7-12, and GCS 13-15.

Variables assessed

Demographic variables (age and sex), mechanism of injury (assault, fall, or road traffic accident (RTA)), CT scan findings, and clinical outcomes were recorded for all patients. The association between each variable and EPTS incidence was analyzed.

Statistical analysis

Data were analyzed using IBM SPSS Statistics for Windows, Version 29 (Released 2022; IBM Corp., Armonk, New York, United States). Both descriptive and inferential statistics were analyzed, as applicable. The chi-square test was used to analyze qualitative data, and significance was ascertained.

Ethical considerations

The study was conducted in accordance with the Declaration of Helsinki and the applicable institutional ethical guidelines. Patient confidentiality was maintained throughout the study.

## Results

Overall incidence

Of the 1,035 patients enrolled, 29 (2.8%) developed at least one EPTS within the first seven days of injury. Among patients with loss of consciousness (n=681), 2.7% (n=18) developed EPTS, and 63.3% (n=18) of all patients with seizures had a history of loss of consciousness.

Seizure incidence by GCS score

**Figure 2:**
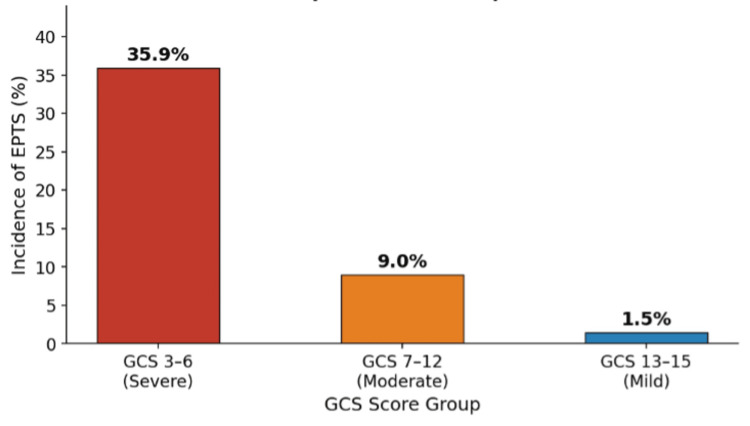
Bar chart illustrating the EPTS incidence (%) across the three GCS score groups. Severe TBI (GCS 3-6) carried the highest risk (35.9%). EPTS: early post-traumatic seizures; GCS: Glasgow Coma Scale; TBI: traumatic brain injury

The patients were classified into three GCS groups. The incidence of EPTS was highest in the severe TBI group (GCS 3-6: 35.9%; 5/14 patients), followed by the moderate group (GCS 7-12: 9.0%; 11/122 patients), and lowest in the mild group (GCS 13-15: 1.5%; 13/899 patients). Severe head injury was significantly associated with EPTS (p<0.001). The findings are summarized in Table 1 and illustrated in Figure 2.

**Table 1:** Incidence of EPTS according to Glasgow Coma Scale (GCS) score group. EPTS: early post-traumatic seizures

GCS Score Group	Total Patients	Patients With EPTS	Incidence (%)
GCS 3-6 (Severe)	14	5	35.9
GCS 7-12 (Moderate)	122	11	9.0
GCS 13-15 (Mild)	899	13	1.5
Total	1035	29	2.8

Seizure incidence by mechanism of injury

**Figure 3:**
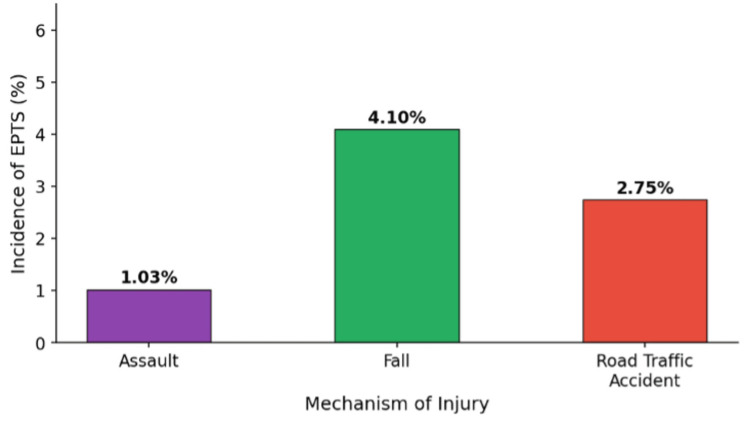
Bar chart comparing EPTS incidence (%) according to mechanism of injury. Falls were associated with the highest incidence of injury. EPTS: early post-traumatic seizures

Falls were the most common mechanism associated with EPTS (4.09%; 15/366), followed by RTAs (2.74%; 13/473) and assault (1.03%; 2/195). The data are presented in Table 2 and Figure 3.

**Table 2:** Incidence of early post-traumatic seizures (EPTS) according to mechanism of injury.

Mechanism of Injury	Total Patients	Patients With EPTS	Incidence (%)
Assault	195	2	1.03
Fall	366	15	4.09
Road Traffic Accident	473	13	2.74

Seizure incidence by CT findings

**Figure 4:**
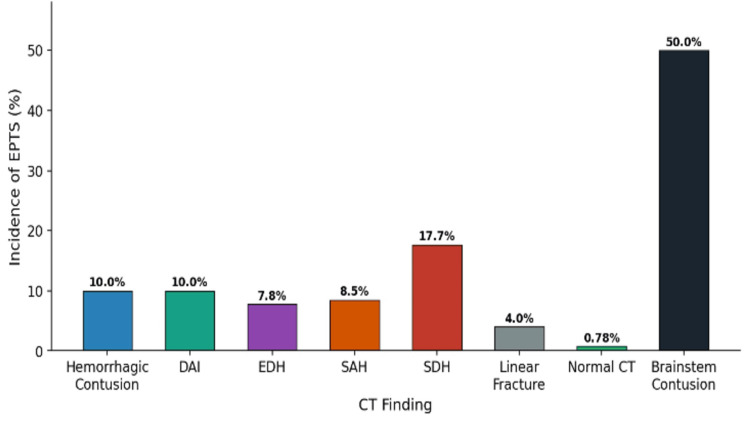
Bar chart showing the EPTS incidence (%) according to CT scan findings. Brainstem contusion and subdural hemorrhage conferred the highest risks. EPTS: early post-traumatic seizures; DAI: diffuse axonal injury; EDH: epidural hematoma; SAH: subarachnoid hemorrhage; SDH: subdural hematoma

The CT findings associated with the highest EPTS risk were brainstem contusion (50.0%; 1/2), subdural hemorrhage (17.7%; 8/45), hemorrhagic contusion (10.0%; 7/70), diffuse axonal injury (10.0%; 1/10), and subarachnoid hemorrhage (8.5%; 3/35). Extradural hemorrhage was associated with a 7.8% risk (3/38), whereas a normal CT scan was associated with a low incidence of 0.78% (6/768). Depressed skull fractures, pneumocephalus, and intraparenchymal hemorrhage were not associated with EPTS in this cohort. Detailed data are presented in Table 3 and Figure 4.

**Table 3:** Incidence of early post-traumatic seizures (EPTS) according to CT scan findings.

CT Finding	Total Patients	Patients With EPTS	Incidence (%)
Hemorrhagic Contusion	70	7	10.0
Diffuse Axonal Injury	10	1	10.0
Depressed Skull Fracture	16	0	0
Extradural Hemorrhage	38	3	7.8
Linear Skull Fracture	50	2	4.0
Normal CT Scan	768	6	0.78
Pneumocephalus	4	0	0
Subarachnoid Hemorrhage	35	3	8.5
Subdural Hemorrhage	45	8	17.7
Intraparenchymal Hemorrhage	4	0	0
Brainstem Contusion	2	1	50.0

Age distribution

**Figure 5:**
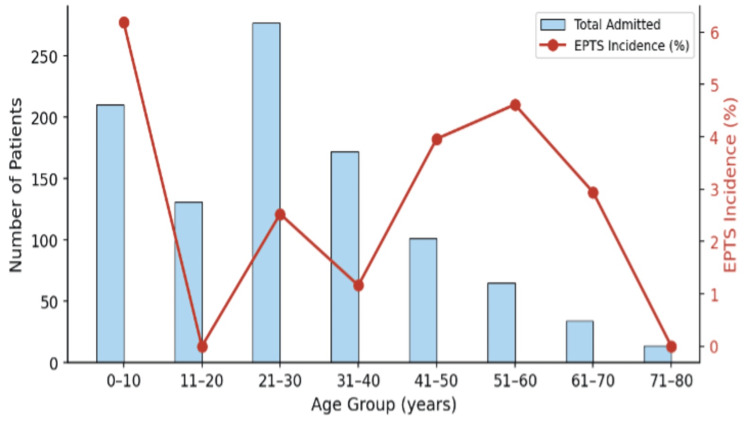
Combined bar-line chart showing the total number of patients admitted (bars) and EPTS incidence percentage (line) across age groups. Children aged 0-10 years had the highest EPTS burden. EPTS: early post-traumatic seizures

The highest absolute number of EPTS cases occurred in the 0-10 year age group (13/210; 6.2%), followed by the 21-30 year age group (7/277; 2.5%). No seizures were recorded in the 11-20 and 71-80 year age groups. Age-wise data are presented in Table 4 and Figure 5, respectively.

**Table 4:** Age-wise distribution of patients and incidence of early post-traumatic seizures (EPTS).

Age Group (Years)	Total Admitted	Patients With EPTS	Incidence (%)
0-10	210	13	6.2
11-20	131	0	0.0
21-30	277	7	2.5
31-40	172	2	1.2
41-50	101	4	4.0
51-60	65	3	4.6
61-70	34	1	2.9
71-80	14	0	0.0

Gender distribution

**Figure 6:**
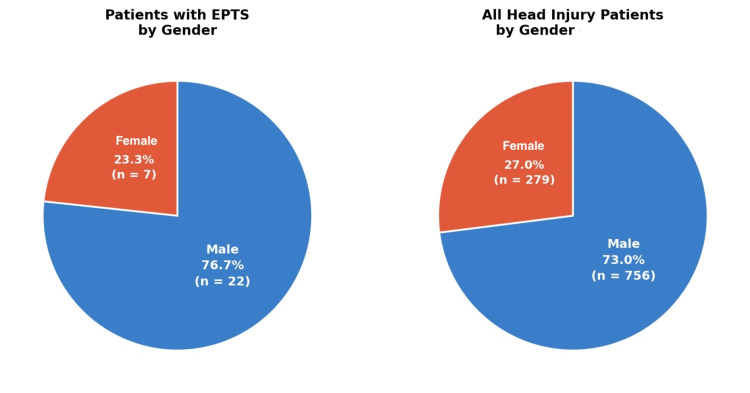
Pie charts comparing the sex distribution between patients with EPTS (left) and the overall head injury population (right). EPTS: early post-traumatic seizures

Male patients constituted 22 out of 29 (76.7%) of EPTS cases, whereas female patients accounted for 23.3% (7). This male predominance was consistent with the overall male predominance in the cohort of patients with head injuries. The gender distribution is shown in Figure 6.

Statistical analysis

Chi-square analysis demonstrated a strong association between the severity of TBI (GCS category) and the occurrence of EPTS (χ^2^=79.05, p<0.001). Similarly, CT scan findings were significantly associated with seizure occurrence (χ^2^=83.87, p<0.001), with intracranial hemorrhagic lesions conferring a higher risk (Table 5).

**Table 5:** Chi-square test results. χ^2^: Pearson's chi-square; dF: degrees of freedom; Sig.: asymptotic significance (two-sided); GCS: Glasgow Coma Scale * Statistically significant at p<0.05.

Variable	χ^2^ value	dF	Asymptotic Sig. (Two-Sided)
GCS Category	79.05	2	<0.001*
Mechanism of Injury	4.34	2	0.114
Age Group	15	7	0.036
CT Findings	83.87	10	<0.001*
Significance Threshold: p<0.05			

Age group also showed a statistically significant association with EPTS (χ^2^=15.00, p=0.036), indicating increased susceptibility in younger patients. However, the mechanism of injury (assault, fall, or RTA) was not significantly associated with seizure occurrence (χ^2^=4.34, p=0.114).

## Discussion

In this prospective observational study of 1,035 patients with head injury at a tertiary neurosurgical center in India, the overall incidence of EPTS was 2.8% (29/1,035). This figure is consistent with lower-range estimates reported in global civilian cohorts (1-12%) and is notably lower than pediatric-specific series, which may reflect the mixed-age composition of our cohort.

The strong correlation between TBI severity and EPTS incidence - with 35.9% (5/14) incidence in the severe group (GCS 3-6) compared to 1.5% (13/899) in mild TBI - is concordant with the published literature. Wiedemayer et al. reported a 5.8% overall EPTS rate with a gradient across severity groups, though they notably found higher EPTS rates in moderate than in severe TBI [10]. In contrast, our data demonstrated a more expected linear increase in injury severity. Similarly, Oluwole found that severity was among the strongest predictors of EPTS [16]. All of our severe head injury patients received anti-epileptic medication, yet 35.9% of them had an episode of EPTS, highlighting the refractory nature of these cases.

The highest EPTS risk in our CT data was observed in brainstem contusion (1, 50%), followed by subdural hemorrhage (8, 17.7%) and hemorrhagic contusion (7, 10%). These findings align with the extensively documented role of intracranial hematomas, particularly subdural hematomas, as the primary structural risk factor for EPTS. Jennett reported a 25% incidence in patients with intracranial hematomas, with intradural lesions conferring a greater risk than extradural lesions [1]. Englander et al. similarly highlighted subdural hematoma with evacuation and multiple cortical contusions as key predictors of poor outcomes [9]. However, EPTS was documented in only one patient with brainstem contusion; the percentage number of 50% is misleading, as the sample size is only two. Larger prospective studies are required to validate this finding. Also, one needs to be alerted to the findings of a normal CT scan. Out of 768 patients with a normal scan, six (0.78%) still developed an EPTS, highlighting the need for continued surveillance and risk counselling.

The preponderance of EPTS in children aged 0-10 years (13, 6.2%) was consistent with the findings of multiple pediatric-focused studies. Asikainen et al. found the highest EPTS rates in children aged ≤7 years (30.8%), attributing this to the heightened vulnerability of the developing brain [13]. Liesemer et al. identified age less than two years as an independent EPTS predictor [11], and Ong et al. highlighted age less than two years and loss of consciousness exceeding 24 hours as key factors in children [12]. Brain immaturity, with heightened excitability and reduced inhibitory tone, likely underlies the greater susceptibility of young children to EPTS. The findings of no EPTS in the age group of 11 to 20 are mostly a random finding due to the small sample size. Whether this is of clinical relevance, perhaps due to the resilience of the younger brains, needs further evaluation in future studies.

Falls were the mechanism with the highest EPTS incidence in our cohort (15, 4.09%), exceeding that of RTAs (13, 2.74%). This may reflect the age distribution of fall-related injuries, with pediatric and elderly patients - both groups with potentially higher seizure susceptibility - more commonly sustaining fall-related TBI. This finding is of public health importance in India, as falls are often neglected in relation to RTAs, but ultimately carry a high risk of complications such as EPTS.

The male predominance, 22 (76.7%) in EPTS cases, reflects the well-established male predisposition to head injury and is consistent with the broader TBI literature. Lee et al. reported a male-to-female ratio of 2.7:1 in their EPTS cohort [14], while Wiedemayer et al. identified male sex as a weaker but significant risk factor for PTS [10].

Regarding prophylactic anticonvulsants, current guidelines recommend phenytoin for the prevention of EPTS in high-risk patients, but only within the first seven days post-injury. Prophylaxis beyond this window has not demonstrated benefits in reducing late seizure development or improving overall outcomes [23,24]. Our findings support targeted prophylaxis in patients with severe TBI, subdural hemorrhage, brainstem contusion, hemorrhagic contusion, and those in younger age groups.

This study had several limitations. First, seizure monitoring was confined to the in-hospital period (up to seven days post-injury), with no EEG confirmation, potentially leading to the underestimation of subclinical or non-convulsive seizures. Second, the single-center design of a tertiary neurosurgical referral institute may not be representative of the broader Indian TBI population, limiting generalizability. Third, certain CT finding subgroups (e.g., brainstem contusion, n=2; diffuse axonal injury, n=10) had very small sample sizes, making the associated EPTS incidence estimates statistically fragile and requiring careful interpretation.

## Conclusions

EPTS occurred in 2.8% (29/1035) of head injury patients in this prospective series from a tertiary neurosurgical center in India. The severity of TBI (p<0.001), a positive hemorrhagic finding in CT scan (p<0.001), and a younger age group (0-10 years) (p=0.036) were statistically significantly associated with EPTS. Falls were the mechanism most strongly associated with EPTS in our study population. CT finding of brainstem contusion was most strongly associated with EPTS; however, this finding might be misleading in view of the small sample size.

These findings support heightened clinical vigilance and early prophylactic anticonvulsant use in high-risk TBI subgroups, specifically in those with severe head injury, intracranial hemorrhage, and younger age. Larger multicenter prospective studies with standardized protocols and extended follow-ups are needed to further characterize the EPTS risk in the Indian population and evaluate optimal prophylactic strategies.

## References

[1] Jennett B (1973). Epilepsy after non-missile head injuries. Scott Med J.

[2] Annegers JF, Hauser WA, Coan SP, Rocca WA (1998). A population-based study of seizures after traumatic brain injuries. N Engl J Med.

[3] Salazar AM, Jabbari B, Vance SC, Grafman J, Amin D, Dillon JD (1985). Epilepsy after penetrating head injury. I. Clinical correlates: a report of the Vietnam Head Injury Study. Neurology.

[4] Frey LC (2003). Epidemiology of posttraumatic epilepsy: a critical review. Epilepsia.

[5] Haltiner AM, Newell DW, Temkin NR, Dikmen SS, Winn HR (1999). Side effects and mortality associated with use of phenytoin for early posttraumatic seizure prophylaxis. J Neurosurg.

[6] Agrawal A, Timothy J, Pandit L, Manju M (2006). Post-traumatic epilepsy: an overview. Clin Neurol Neurosurg.

[7] Christensen J, Pedersen MG, Pedersen CB, Sidenius P, Olsen J, Vestergaard M (2009). Long-term risk of epilepsy after traumatic brain injury in children and young adults: a population-based cohort study. Lancet.

[8] Temkin NR (2003). Risk factors for posttraumatic seizures in adults. Epilepsia.

[9] Englander J, Bushnik T, Duong TT (2003). Analyzing risk factors for late posttraumatic seizures: a prospective, multicenter investigation. Arch Phys Med Rehabil.

[10] Wiedemayer H, Triesch K, Schäfer H, Stolke D (2002). Early seizures following non-penetrating traumatic brain injury in adults: risk factors and clinical significance. Brain Inj.

[11] Liesemer K, Bratton SL, Zebrack CM, Brockmeyer D, Statler KD (2011). Early post-traumatic seizures in moderate to severe pediatric traumatic brain injury: rates, risk factors, and clinical features. J Neurotrauma.

[12] Ong LC, Dhillon MK, Selladurai BM, Maimunah A, Lye MS (1996). Early post-traumatic seizures in children: clinical and radiological aspects of injury. J Paediatr Child Health.

[13] Asikainen I, Kaste M, Sarna S (1999). Early and late posttraumatic seizures in traumatic brain injury rehabilitation patients: brain injury factors causing late seizures and influence of seizures on long-term outcome. Epilepsia.

[14] Lee ST, Lui TN (1992). Early seizures after mild closed head injury. J Neurosurg.

[15] Lee ST, Lui TN, Chang CN, Cheng WC, Wang DJ, Heimburger RF, Lin CG (1989). Prophylactic anticonvulsants for prevention of immediate and early postcraniotomy seizures. Surg Neurol.

[16] Oluwole OS (2011). Incidence and risk factors of early post-traumatic seizures in Nigerians. Brain Inj.

[17] Povlishock JT, Katz DI (2005). Update of neuropathology and neurological recovery after traumatic brain injury. J Head Trauma Rehabil.

[18] Vespa PM, Nuwer MR, Nenov V (1999). Increased incidence and impact of nonconvulsive and convulsive seizures after traumatic brain injury as detected by continuous electroencephalographic monitoring. J Neurosurg.

[19] Willmore LJ, Rubin JJ (1981). Antiperoxidant pretreatment and iron-induced epileptiform discharges in the rat: EEG and histopathologic studies. Neurology.

[20] Ikeda A, Shibasaki H, Nagamine T (1996). Dissociation between seizure-associated runaway and interictal epileptiform discharges. Epilepsia.

[21] Patel M (2004). Mitochondrial dysfunction and oxidative stress: cause and consequence of epileptic seizures. Free Radic Biol Med.

[22] Bhatt DL, Fox KA, Hacke W (2006). Clopidogrel and aspirin versus aspirin alone for the prevention of atherothrombotic events. N Engl J Med.

[23] (2007). Guidelines for the management of severe traumatic brain injury. J Neurotrauma.

[24] Temkin NR, Dikmen SS, Wilensky AJ, Keihm J, Chabal S, Winn HR (1990). A randomized, double-blind study of phenytoin for the prevention of post-traumatic seizures. N Engl J Med.

